# Branched-chain amino acids alleviate sepsis-induced myocardial dysfunction via inhibition of protein tyrosine phosphatase-6

**DOI:** 10.1038/s41392-025-02224-9

**Published:** 2025-04-30

**Authors:** Shu-Rui Pang, Yu-Tong Zhu, Hou-Zao Chen, Jian-Fei Pei, De-Pei Liu

**Affiliations:** State Key Laboratory of Common Mechanism Research for Major Diseases, Department of Biochemistry and Molecular Biology, Institute of Basic Medical Sciences, Chinese Academy of Medical Sciences and Peking Union Medical College, Beijing, China

**Keywords:** Cardiology, Cardiovascular diseases

**Dear editor**,

Sepsis-induced myocardial dysfunction (SIMD) is a distinct type of heart failure (HF) that differs from other forms of HF in its rapid onset, immune storm-driven, and accompanied muti-organ dysfunction. Although SIMD dramatically increases the mortality rate of patients with sepsis from 10% to 40–70%,^1^ effective pharmacotherapies remain limited. Defects in branched-chain amino acid (BCAA) catabolism and the accumulation of BCAA have been recognized as metabolic hallmarks of HF induced by ischemic injury, hypertension, diabetes, and so on. However, BCAAs appear to play an opposite role in SIMD: serum BCAA levels are negatively correlated with disease severity in patients^2^; a BCAA-rich mixture has been shown to preserve systolic function and improve coronary flow in isolated septic rat hearts.^3^ Given these paradoxical findings, we investigated the potential cardioprotective role of BCAA in SIMD to improve the current understanding of the disease and offer new perspectives on promising targeted therapies.

SIMD in mice was induced by an intraperitoneal injection of 20 mg/Kg lipopolysaccharide (LPS), a simple, reproductive, and highly standardized model. Twenty-four hours later, BCAA levels in the heart and serum decreased in LPS-injected mice (data not shown), suggesting the involvement of BCAAs and their distinct mechanisms in SIMD. To investigate the effects of BCAAs on SIMD, 8-week-old mice were fed a BCAA-enriched diet (BCAAe diet, containing 50% more BCAA) for 0, 2 or 6 weeks (w). Supplementation for 6 w, but not 2 w, increased cardiac BCAA content by 20% (data not shown). This increase was coupled with a reduced mortality rate in SIMD mice (from 50% to 19.8%) (Fig. 1a). Echocardiographic analysis revealed improved cardiac systolic function in SIMD mice receiving 6 w of BCAA supplementation, as indicated by elevated ejection fraction (EF), fractional shortening (FS) and cardiac output (Fig. 1a). Additionally, 6 w of BCAA supplementation effectively reduced the LPS-induced increase in serum Troponin I levels (Fig. 1a).

**Fig. 1:**
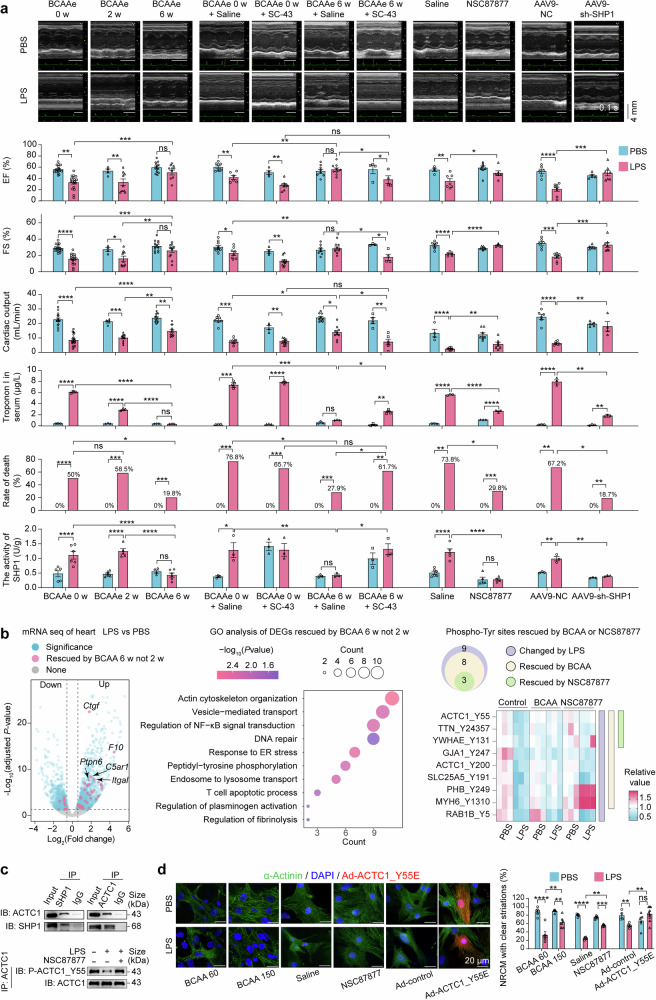
BCAAs inhibit *Ptpn6/*SHP1 to maintain ACTC1_Y55 phosphorylation, thereby preserving cardiac filament stability against SIMD. **a** Analysis of cardiac function, troponin I level, rate of death and cardiac *Ptpn6/*SHP1 activity of SIMD mice with BCAA supplementation and *Ptpn6/*SHP1 modulation. Eight-week-old mice were fed a BCAAe diet for 0, 2, or 6 weeks prior to LPS injection. SC-43 (*Ptpn6/*SHP1 agonist, 30 mg/kg) or NSC87877 (*Ptpn6/*SHP1 inhibitor, 5 mg/Kg) was administered 30 min after LPS injection. AAV9 (5 × 10^11^ vector genomes per mouse) was injected 4 weeks before LPS injection. Representative images of echocardiography (scale bar: 4 mm, 0.1 s). Left ventricle systolic function, expressed as ejection fraction (EF) (%), fractional shortening (FS) (%), and cardiac output (mL/min), was evaluated 24 h after LPS injection (*n* = 3~21 per group). Troponin I in serum (*n* = 3~5 per group), rate of death (*n* = 4~24 per group) and cardiac *Ptpn6/*SHP1 activity (n = 3 ~ 6 per group) were also assessed at 24 h after LPS injection. **b** Bioinformatics analysis of RNA sequencing and phosphorylation mass spectrometry data. Left panel: volcano plot from RNA sequencing illustrates differentially expressed genes (DEGs, shown in blue) between healthy and SIMD mouse hearts, using a threshold of |log2 (foldchange)| > 0.58 and an adjusted *P*-value < 0.05 (*n* = 3 per group). DEGs that were restored by 6 w (but not 2 w) of BCAA supplementation are highlighted in pink, with *Ptpn6*/SHP1 ranking fourth. Middle panel: Gene Ontology (GO) analysis of DEGs restored by BCAA 6 w (but not 2 w) revealed significantly enriched biological process terms. The Term “Actin cytoskeleton organization” is associated with ACTC1, while “Peptidyl−tyrosine phosphorylation” pertains to Tyr phosphorylation. Other enriched terms reflect known changes during sepsis development. Right panel: Venn diagram and heatmap from phospho-MS illustrate differentially phosphorylated sites between healthy and SIMD mouse hearts, using a threshold of |log2 (foldchange)| > 0.263 and an adjusted *P*-value < 0.05 (*n* = 3 per group). Nine differentially phosphorylated sites induced by LPS are shown in purple. Among these, eight were reversed by BCAA supplementation (displayed in yellow), and three were reversed by NSC87877 (displayed in green). **c** Representative immunoblot (IB) images. Mouse hearts were homogenized, lysed, and subjected to immunoprecipitation (IP). Upper panel: analysis of the interaction between *Ptpn6*/SHP1 and ACTC1. Lower panel: analysis of ACTC1_Y55 phosphorylation in response to LPS and NSC87877 treatment. The blots shown are representative of three independent experiments. **d** Representative images and percentage of NRCMs with clear filament striations in response to LPS (1 μg/mL), BCAA (60 or 150 mg/L), NSC87877 (0.5 μg/mL) and Ad-ACTC1_Y55E. Immunofluorescence staining with anti‑α‑Actinin was used to visualize NRCM filament striations. Left panel: representative images of NRCM filaments (scale bar, 20 μm). Right panel: bar plot showing the percentage of NRCMs with clear striations. All cell experiments were repeated three times, and values are expressed as mean ± SEM. Statistical significance is indicated as follows: *P < 0.05, **P < 0.01, ***P < 0.001, ****P < 0.0001. A Chi-square test was used to analyze the rate of death data in **a**. Two-way analysis of variance (ANOVA) with a Bonferroni post hoc test was performed for statistical analysis in **a**, **d**. PBS phosphate-buffered saline, LPS lipopolysaccharide, BCAAe BCAA-enriched diet, 0/2/6 w BCAA supplementation through diet for 0, 2 or 6 weeks, AAV9 adeno associated virus 9, phospho-MS phosphorylation mass spectrometry, NRCM neonatal rat cardiomyocytes, Ad adenovirus, Tyr tyrosine, Y tyrosine, E glutamic acid, P phosphorylation

RNA sequencing was performed to investigate the mechanism by which BCAAs protect SIMD mice. Forty-eight differentially expressed genes (DEGs, shown in pink) in the mouse heart were altered by LPS but restored by 6 w (rather than 2 w) of BCAA supplementation (Fig. 1b). Among these DEGs, protein tyrosine phosphatase-6 (PTPN6, or Src homology region 2 domain-containing phosphatase-1 (SHP1)), which is known to exert oxidative, apoptotic, and anti-angiogenic effects in cardiac diseases, ranked fourth. Gene ontology (GO) analysis revealed significant enrichment of tyrosine phosphorylation in biological process terms, suggesting that the tyrosine phosphatase *Ptpn6*/SHP1 is likely an important downstream target of BCAAs. Consistently, the enzymatic activity of cardiac *Ptpn6*/SHP1 was increased by LPS but reduced by 6 w of BCAA supplementation (Fig. 1a). Phosphorylation mass spectrometry (phospho-MS) analysis showed a decrease in phosphorylated tyrosine (Tyr) following LPS treatment, while serine and threonine phosphorylation remained unchanged (data not shown).

To investigate the effect of *Ptpn6*/SHP1 on SIMD, its agonist (SC-43), inhibitor (NSC87877) and AAV9 (adeno-associated virus, mediated knockdown of *Ptpn6/*SHP1) were used. SC-43 effectively activated *Ptpn6/*SHP1 and blocked the positive effect of BCAAs against SIMD, as evidenced by poor cardiac systolic function and a lower survival rate (Fig. 1a). Both NSC87877 and AAV9-sh-SHP1 reduced *Ptpn6*/SHP1 activity, protecting the survival and cardiac systolic function of SIMD mice (Fig. 1a).

Phospho-MS identified 9 Tyr sites in cardiac proteins that were altered by LPS, and all these sites were downregulated (Fig. 1b). Among these 9 sites, 8 were rescued by BCAA and 3 were rescued by NSC87877 (Fig. 1b). In conclusion, three sites were rescued by both BCAAs and NSC87877, and two of these sites are located in proteins that form muscle filaments (ACTC1 and TTN). Consistently, immunofluorescence staining of α‑Actinin in NRCMs (neonatal rat cardiomyocytes), a commonly used primary cardiomyocyte model,^4^ revealed that LPS-induced damage to muscle filaments, resulting in the loss of clear striations, and this damage was prevented by both BCAAs and NSC87877 (Fig. 1d). Among these Tyr sites, ACTC1_Y55 attracted our attention because it’s highly conserved and modulates actin structure and filament function. Additionally, the “Actin cytoskeleton organization” term was significantly enriched in the GO analysis (Fig. 1b). Immunoprecipitation experiments revealed that ACTC1 co‑precipitated with *Ptpn6*/SHP1, and its phosphorylation level was decreased by LPS but restored by NSC87877 (Fig. 1c), demonstrating that *Ptpn6/*SHP1 interacted directly with ACTC1 and dephosphorylated it upon LPS stimulation. To mimic phosphorylated ACTC1, the adenovirus Ad‑ACTC1‑Y55E (E: glutamic acid) was generated. Immunofluorescence imaging showed that Ad‑ACTC1‑Y55E preserved NRCM filaments with clear striations in the presence of LPS, similar to the effects observed with BCAA and NSC87877 (Fig. 1d). The observation that cardiac filament integrity was maintained by BCAA, NSC87877 and ACTC1_Y55E is consistent with previous findings that a BCAA‑rich mixture can preserve systolic properties in isolated septic rat hearts.^3^

Collectively, dietary BCAA supplementation maintained ACTC1_Y55 in a phosphorylated state by inhibiting *Ptpn6*/SHP1, resulting in protective cardiac function against SIMD. Notably, BCAAs were found to exert an effect in SIMD that is opposite to their role in other forms of heart failure. The BCAA‑SHP1‑tyrosine phosphorylation axis, reported here for the first time, updates the traditional view that BCAAs protect against SIMD by providing energy. Together with previous findings showing that serine phosphorylation of SERCA regulates cardiac diastolic function in SIMD,^5^ these results suggest that alterations in the phosphorylation levels of cardiac proteins play a crucial role in the pathogenesis of SIMD. Mechanistically, Tyr phosphorylation, particularly in filament proteins, offers new insights into heart failure development. We acknowledge that these mechanisms would be more clinically relevant if validated in a sepsis model induced by cecal ligation and puncture. Taken together, our results suggest that SHP1 might be a potential target for SIMD treatment.

## Supplementary information

Materials and Methods

## Data Availability

RNA sequencing data are publicly available from the China National Center for Bioinformation (submission number subCRA022712). The mass spectrometry proteomics data have been deposited to the ProteomeXchange Consortium (https://proteomecentral.proteomexchange.org) via the iProX partner repository with the dataset identifier PXD062235.
