# Supplementary material for: Branched-chain amino acids alleviate sepsis-induced myocardial dysfunction via inhibition of protein tyrosine phosphatase-6
Source: Signal Transduct Target Ther. 2025 Apr 30;10:135. doi: 10.1038/s41392-025-02224-9 (PMC12041460; doi:10.1038/s41392-025-02224-9)
Supplement: Supplementary file 1 — Materials and Methods [file 41392_2025_2224_MOESM1_ESM.docx]

Supplementary Materials for

Branched-chain amino acids alleviate sepsis-induced myocardial dysfunction via inhibition of protein tyrosine phosphatase-6

Shu-Rui Pang 1#, Yu-Tong Zhu 1#, Hou-Zao Chen 1, Jian-Fei Pei 1*, De-Pei Liu 1*

Correspondence to: liudp@pumc.edu.cn

**This PDF file includes:**

Materials and Methods

Materials and Methods

Mouse LPS infection and feeding protocols

All animal experiments were approved by the Animal Care and Use Committee of the Institute of Basic Medical Sciences, the Laboratory Animal Ethics Committee, and the Laboratory Animal Center of the Chinese Academy of Medical Sciences and Peking Union Medical College (Approval No. ACUC-A02-2023-055).

Eight-week-old male C57BL/6J mice (23 ± 2 g) were housed in a temperature-controlled (25 °C), specific pathogen-free environment with free access to water and food. The mice were randomly assigned to three groups and subjected to different feeding regimens:

Control group: Standard diet (STD diet, HFK Bioscience Co., LTD., Beijing) for 6 weeks.

Short-term BCAA diet group: STD diet for 4 weeks, followed by a branched-chain amino acid-enriched (BCAA) diet (HFK Bioscience Co., LTD., Beijing) for 2 weeks.

Long-term BCAA diet group: BCAA diet for 6 weeks.

After the 6-week feeding period, a sepsis-induced myocardial dysfunction (SIMD) model was established by intraperitoneal injection of lipopolysaccharide (LPS, 20 mg/kg; Escherichia coli O55:B5, Sigma) or an equivalent volume of phosphate-buffered saline (PBS) as a control. Various physiological parameters were recorded 24 hours post-injection.

SHP1 inhibitors and agonists

NSC87877 (Santa Cruz Biotechnology, sc-204139) or saline (PBS) was administered to mice via intraperitoneal injection (5 mg/kg) 30 minutes after LPS stimulation. In parallel, NSC87877 was added to the culture medium of neonatal rat cardiomyocytes (NRCMs) at a final concentration of 0.25 μg/ml, 30 minutes after LPS stimulation (1 μg/ml).

Similarly, SC-43 (MCE, HY-136657) or its vehicle control (DMSO: corn oil, 1:9) was administered to mice via intraperitoneal injection (30 mg/kg) 30 minutes after LPS stimulation. For in vitro experiments, SC-43 was added to the NRCM culture medium at a final concentration of 1.5 μg/ml, 30 minutes after LPS stimulation (1 μg/ml).

Virus

AAV9-sh-SHP1, designed for mSHP1 knockdown, and its control vector AAV9-U6-GFP (AAV9-NC) were obtained from Vigene Biosciences. Eight-week-old C57BL/6J mice were injected via the tail vein with AAV9 (5 × 10¹¹ vector genomes (vg) per mouse, diluted in 200 µl PBS). Four weeks after viral administration, mice were subjected to LPS injection.

For in vitro experiments, Ad-control and Ad-ACTC1_Y55E were constructed using the pDC316-eGFP vector and the PCMV-Flag+His-P2A-GFP vector. NRCMs (5 × 10⁵ cells per well) were seeded in 24-well plates. The following day, the designated virus was added to the culture medium (MOI = 30) without antibiotics. After 12 hours, the cells were washed twice with PBS and maintained in growth medium for an additional 36 hours. Then, cells were treated with either LPS or PBS. After 12 hours, cells were harvested for immunofluorescence analysis.

Echocardiography

Transthoracic echocardiography was performed 24 hours after intraperitoneal LPS injection. Mice were anesthetized with inhaled isoflurane (3% for induction and 1% for maintenance), and cardiac function was assessed using a preclinical ultrasound system (Vevo 1100, FUJIFILM Visual Sonics, Canada) equipped with a 30 MHz linear array transducer. Cardiac function (such as ejection fraction (EF) and fractional shortening (FS)) were analyzed.

BCAA concentration analysis

The BCAA levels in the heart were measured using a commercially available kit. Heart tissues were homogenized, centrifuged, and the supernatant was collected. The BCAA concentration was then quantified using a BCAA assay kit (Sigma, MAK003) following the manufacturer instructions.

Protein tyrosine phosphatase nonreceptor type 6 (ptpn6) activity analysis

Heart tissues were homogenized and centrifuged, and then, the supernatant was collected to measure ptpn6 activity using a RediPlate 96 EnzChek Tyrosine Phosphatase Assay Kit (Thermo Fisher, R-22067) according to the manufacturer's instructions. The fluorescence intensity of each well was measured at 358/452 nm every five minutes for a total of 90 minutes at 37 °C in the dark.

Western blot analysis

Heart tissue samples were collected, and total protein was extracted. Protein samples (20 μg) were separated by sodium dodecyl sulfate–polyacrylamide gel electrophoresis (SDS-PAGE) and subsequently transferred onto a polyvinylidene fluoride (PVDF) membrane. After blocking with 5% fat-free milk at room temperature for 1 hour, the membrane was incubated overnight at 4 °C with primary antibodies against SHP1 (1:200, ab227503, Abcam), phospho-tyrosine (1:200, PTM-703, Jingjie Biotechnology Co., LTD., Hangzhou), GAPDH (1:1000, ab8245, Abcam), and ACTC1 (1:200, ab46805, Abcam).

The membrane was then washed three times with Tris-buffered saline containing Tween-20 (TBST) and incubated with horseradish peroxidase (HRP)-conjugated secondary antibodies at room temperature for 1.5 hours. After additional washes, the membrane was treated with Immobilon Western chemiluminescent HRP substrate (Beyotime, Shanghai), and the signals were detected using an electrochemiluminescence (ECL) method as previously described. Protein expression levels were normalized to GAPDH.

RNA sequencing analysis

Transcriptional sequence analysis was conducted in collaboration with IGENECODE (Beijing). Mice were fed either a standard diet (STD) or a branched-chain amino acid-enriched (BCAAe) diet (as described in Fig. a) and subsequently injected with LPS (20 mg/kg) or saline. Heart tissues were collected 24 hours after LPS stimulation for RNA sequencing (RNA-seq) analysis. Total mRNA was extracted from three mice per group, resulting in a total of 18 samples across the following conditions: control diet with saline, control diet with LPS, BCAAe diet for 2 weeks with saline, BCAAe diet for 2 weeks with LPS, BCAAe diet for 6 weeks with saline, and BCAAe diet for 6 weeks with LPS. For library preparation, 3 μg of RNA per sample was used as input material. Sequencing libraries were generated using the NEBNext® Ultra RNA Library Prep Kit for Illumina® (NEB, USA) following the manufacturer’s protocol, with index codes assigned to each sample for identification. Clustering of index-coded samples was performed on a cBot cluster generation system using the HiSeq PE Cluster Kit v4-cBot-HS (Illumina) according to the manufacturer’s instructions. Following cluster generation, sequencing was carried out on an Illumina HiSeq 4000 platform, generating 150-bp paired-end reads. Transcript abundance was quantified using fragments per kilobase of exon per million mapped reads (FPKM) values.

Proteomics and phosphorylation mass spectrometry

A proteomics experiment was conducted in collaboration with PTM Bio Inc. (Hangzhou). Mice were fed either a standard diet (STD) or a BCAAe diet for 6 weeks and then injected with LPS (20 mg/kg) or saline, followed by NSC87877 (5 mg/kg) or saline. Heart tissues were collected 24 hours after LPS stimulation for liquid chromatography–mass spectrometry (LC–MS) analysis. Total heart protein was extracted using a lysis buffer containing 8 M urea, 1% protease inhibitor, and 1% phosphatase inhibitor, with ultrasonication. Cell debris was removed by centrifugation at 12,000 × g for 10 minutes at 4 °C, and the protein concentration in the supernatant was determined using a bicinchoninic acid (BCA) assay. Proteins were digested into peptides overnight using trypsin. The peptides were separated using liquid chromatographic mobile phase A and further resolved in a NanoElute ultrahigh-performance liquid chromatography (UHPLC) system. Subsequently, the peptides were injected into a capillary ion source for ionization and analyzed using timsTOF Pro mass spectrometry. To assess protein phosphorylation rates, an additional enrichment step was performed prior to LC–MS analysis.

Statistical analysis

In all experiments, samples were randomized and evaluated by researchers blinded to group allocation. Normally distributed datasets were analyzed using unpaired Student’s t-tests for comparisons between two independent groups, while one-way or two-way ANOVA followed by Bonferroni’s post hoc test was used for multiple comparisons involving at least three groups.

Quantitative data are presented as the mean ± SEM. All statistical analyses were performed using GraphPad Prism 9.0 software. Mortality rates were analyzed using the chi-square test or Fisher’s exact test. For all statistical tests, a P-value < 0.05 was considered statistically significant, and all tests were two-tailed.
